# Validation of quantitative assessment of florbetaben PET scans as an adjunct to the visual assessment across 15 software methods

**DOI:** 10.1007/s00259-023-06279-0

**Published:** 2023-06-10

**Authors:** Aleksandar Jovalekic, Núria Roé-Vellvé, Norman Koglin, Mariana Lagos Quintana, Aaron Nelson, Markus Diemling, Johan Lilja, Juan Pablo Gómez-González, Vincent Doré, Pierrick Bourgeat, Alex Whittington, Roger Gunn, Andrew W. Stephens, Santiago Bullich

**Affiliations:** 1 Life Molecular Imaging GmbH, Berlin, Germany; 2 MIM Software Inc., Cleveland, OH USA; 3 Hermes Medical Solutions, Stockholm, Sweden; 4 Qubiotech Health Intelligence, A Coruña, Spain; 5 Department of Molecular Imaging & Therapy, Austin Health, Melbourne, Australia; 6 CSIRO, Brisbane, Australia; 7 Invicro, London, UK

**Keywords:** Alzheimer’s disease, Amyloid-beta, Florbetaben, Quantification, Centiloid, Mild cognitive impairment

## Abstract

**Purpose:**

Amyloid positron emission tomography (PET) with [^18^F]florbetaben (FBB) is an established tool for detecting Aβ deposition in the brain in vivo based on visual assessment of PET scans. Quantitative measures are commonly used in the research context and allow continuous measurement of amyloid burden. The aim of this study was to demonstrate the robustness of FBB PET quantification.

**Methods:**

This is a retrospective analysis of FBB PET images from 589 subjects. PET scans were quantified with 15 analytical methods using nine software packages (MIMneuro, Hermes BRASS, Neurocloud, Neurology Toolkit, statistical parametric mapping (SPM8), PMOD Neuro, CapAIBL, non-negative matrix factorization (NMF), Amyloid^IQ^) that used several metrics to estimate Aβ load (SUVR, centiloid, amyloid load, and amyloid index). Six analytical methods reported centiloid (MIMneuro, standard centiloid, Neurology Toolkit, SPM8 (PET only), CapAIBL, NMF). All results were quality controlled.

**Results:**

The mean sensitivity, specificity, and accuracy were 96.1 ± 1.6%, 96.9 ± 1.0%, and 96.4 ± 1.1%, respectively, for all quantitative methods tested when compared to histopathology, where available. The mean percentage of agreement between binary quantitative assessment across all 15 methods and visual majority assessment was 92.4 ± 1.5%. Assessments of reliability, correlation analyses, and comparisons across software packages showed excellent performance and consistent results between analytical methods.

**Conclusion:**

This study demonstrated that quantitative methods using both CE marked software and other widely available processing tools provided comparable results to visual assessments of FBB PET scans. Software quantification methods, such as centiloid analysis, can complement visual assessment of FBB PET images and could be used in the future for identification of early amyloid deposition, monitoring disease progression and treatment effectiveness.

**Supplementary Information:**

The online version contains supplementary material available at 10.1007/s00259-023-06279-0.

## Introduction

Alzheimer’s disease (AD) is the leading cause of dementia and constitutes 60–80% of dementia cases over the age of 65 years. There is a protracted preclinical (asymptomatic) period during which abnormal traits manifest in the central nervous system detectable via biomarkers of the disease. It is characterized by the persistent formation of amyloid plaques and the subsequent development of amyloid-dependent tau pathology, neuroinflammation, and synaptic dysfunction, followed by clinical symptoms [1]. As such, the protracted AD continuum is accompanied by increasing severity of symptoms and is ultimately fatal. Detecting and quantifying such biomarkers are critical for early diagnosis of AD, for the stratification of patients for targeted disease-modifying drugs, and to monitor the effects of potential treatments by providing information about the level of relevant neuropathology [2–5].

Biomarkers and methods for AD pathology detection have been established in recent years, which induced a shift towards a biomarker-assisted diagnosis. Guidelines emphasize the fundamental role of amyloid in the AD diagnostic process. Amyloid positron emission tomography (PET) with florbetaben (FBB) is an established tool for detecting Aβ deposition in vivo. FBB underwent a global multicenter development program and was approved by the European Medicines Agency (EMA) and the US Food and Drug Administration (FDA) in 2014, and other national agencies have subsequently approved its use [6, 7]. FBB has been robustly validated against histopathological confirmation of neuritic Aβ plaque density in the brain as the standard of truth (SoT) [8]. Visual inspection of the FBB PET scans is the approved and validated method for image interpretation and has proven to be very reliable in clinical practice [9].

Although visual assessment (VA) is an appropriate method for the vast majority of scans assessed in clinical practice, it has been proposed that certain situations would benefit from additional quantitative information gleaned from the amyloid images [10, 11]. In a heterogeneous clinical population, for example, VA can be challenging, especially for less-experienced readers: anatomical abnormalities, such as cortical thinning caused by atrophy and ventricular enlargement, may hamper VA; the presence of other neurodegenerative disorders can also confound the assessment on a purely visual basis; and the dichotomous readout of VA may lack the required sensitivity to assess longitudinal changes of amyloid load [12, 13]. A substantial part of the clinical population now includes patients with early-stage disease, which may only show isolated regional uptake and emerging amyloid deposits [14, 15]. Additionally, a small fraction of PET images is only assessed with low confidence, e.g., when amyloid levels are intermediate [16, 17]. In such situations, the continuous measurement of amyloid burden with quantification can provide additional information to the binary VA and increase confidence in such equivocal situations.

Indeed, quantification of PET images is commonly performed in research studies [18] and therapeutic clinical trials [3, 19–24]. The recent FDA accelerated approval of lecanemab, an anti-amyloid antibody, was based on the observed reduction of Aβ plaques as quantified and monitored by amyloid PET [25]. Detection of longitudinal changes using quantification methods is established in the research and development setting [14] with many PET software packages capable of calculating amyloid burden both on a composite and a regional level [26, 27].

In this context, this retrospective data analysis was conducted to evaluate FBB PET quantification as an adjunct to VA. This study was aimed at providing scientific evidence of the robustness and additional value of FBB PET quantification. FBB PET scans from previous clinical trials were quantitatively assessed with several analytical methods. The diagnostic performance (i.e., sensitivity and specificity) of quantification against the histopathological confirmation of Aβ load was estimated and compared to the effectiveness of the approved VA method. Additionally, the concordance between visual and quantitative evaluation of FBB PET scans was assessed. The reliability and comparability of the different analytical methods were further tested.

## Methods

### Study design

This is a retrospective analysis of FBB PET images that had been obtained in previous clinical trials. All data had been acquired in accordance with the Declaration of Helsinki and after approval of local ethics committees; the informed consent from subjects included the future scientific analyses of acquired PET scans.

### Participants

The study population consisted of 589 subjects with at least one available FBB PET scan from previously completed clinical studies. All subjects were grouped into 4 cohorts to address different study objectives (Table 1).

**Table 1:** Description of the cohorts included in this retrospective analysis

Cohort	Clinical data source	Clinical diagnosis	Objective for retrospective analysis	Subset analysis	Visual assessment
#1, *n* = 91	NCT01020838 [8, 9]	59 AD 4 DLB 9 DEM 19 NDV	To assess the sensitivity and specificity of quantification methods against the histopathological confirmation		3 expert blinded readers
			To compare the diagnostic performance of quantification methods with previously established visual assessment performed by 3 expert blinded readers and 5 newly trained blinded readers		5 newly trained blinded readers
#2, *n* = 439	Images from clinical phase 1, 2, and 3 studies were visually assessed by five independent blinded readers in a pooled read study	183 eHC 169 AD 9 DLB 11 FTLD 1 FTD 51 MCI 5 PD 4 VaD 6 other	To assess the concordance between visual assessment performed by 5 blinded readers and binary quantitative assessment of FBB PET scans To assess agreement and reliability between (inter-software) and within (intra-software) analytical methods	Subset 1 (*n* = 386): excluded subjects from cohort #1 used to generate the cut-off Subset 2 (*n* = 439): represents the full sample Subset 3 (*n* = 336): based on subset 2 and included only subjects with consensus among 5 independent blinded readers Subset 4 (*n* = 84): randomly selected scans to assess intra-software reliability	5 newly trained blinded readers
#3, *n* = 45	NCT01138111 [28]	45 MCI	To assess the prognostic accuracy of quantitative assessment of amyloid-beta load using FBB PET for progression to AD To assess the capacity of quantitative assessment of amyloid-beta load using FBB PET to detect longitudinal changes		
#4, *n* = 35	Global Alzheimer’s Association Information Network (GAAIN) [29]	10 yHC 6 eHC 9 MCI 8 AD 2 FTD	To compare the results of FBB PET quantification against the quantitative results provided in a publicly available dataset		

*AD* Alzheimer’s disease, *DEM* other dementia, *DLB* dementia with Lewy bodies, *eHC* elderly healthy control, *FTD* frontotemporal dementia, *FTLD* frontotemporal lobar degeneration, *MCI* amnestic mild cognitively impaired, *NDV* non-demented volunteer, *PD* Parkinson’s disease, *VaD* vascular dementia, *yHC* young healthy control

Cohort #1 included end-of-life subjects with histopathological confirmation of presence or absence of Aβ neuritic plaques (*n* = 81) and young healthy controls (*n* = 10). Aβ status in the young healthy controls (27.4 ± 5.5 years (mean ± SD)) was considered absent in all brain regions.

Cohort #2 comprised 439 subjects, including 53 subjects from cohort #1 and 45 subjects from cohort #3 that were visually assessed by 5 independent newly trained blinded readers. Four different subsets were analyzed. Subset 1 excluded subjects used to generate the cut-off and comprised 386 subjects. Subset 2 represents the full sample, consisting of all 439 subjects. Subset 3 was based on subset 2 but included only subjects where the 5 independent blinded readers had consensus in the assessment, resulting in a subset of *n* = 336 subjects. Subset 4 comprised randomly selected scans to assess intra-software reliability.

Cohort #3 included amnestic mild cognitively impaired (MCI) subjects (*n* = 45) that underwent three FBB PET scans at baseline and a 4-year clinical follow-up.

Cohort #4 was the publicly available GAAIN (Global Alzheimer s Association Information Network) dataset (http://www.gaain.org/centiloid-project) (*n* = 35) including FBB PET scans of patients with MCI, AD, and frontotemporal dementia (FTD) and elderly and young healthy controls.

### Image acquisition and reconstruction

The PET image acquisition and reconstruction were performed as detailed in previous literature [8, 9]. Briefly, all subjects underwent a 20-min PET scan (4 × 5-min dynamic frames) starting at 90 min after intravenous injection of 300 MBq ± 20% of FBB. PET scans were then reconstructed using the ordered subset expectation maximization (OSEM) algorithm with 4 iterations and 16 subsets (zoom = 2) or comparable reconstruction. Corrections were applied for attenuation, scatter, randoms, and dead time. 3D volumetric T1-weighted brain MRI data was available for 497 subjects.

### Post-mortem histopathology

Histopathological confirmation of Aβ presence or absence in the brain was available for subjects from cohort #1. Aβ was regarded as present in a given brain region when moderate or frequent plaques were present either by Bielschowsky silver staining (BSS) or immunohistochemistry. Detailed autopsy procedures and collection/analysis of these post-mortem histopathology specimens are described in detail in [8, 9].

### Visual assessment

FBB PET scans from cohort #1 were visually assessed by 3 independent and blinded expert readers and 5 newly trained, independent blinded readers. PET scans from cohort #2 were visually assessed by independent blinded readers, which were not identical to those that assessed cohort #1. All blinded readers followed the same reading methodology as previously described by Seibyl et al. [9]. No structural information from CT or MRI was available to interpreters.

### Quantification of amyloid load in PET images

**Fig. 1:**
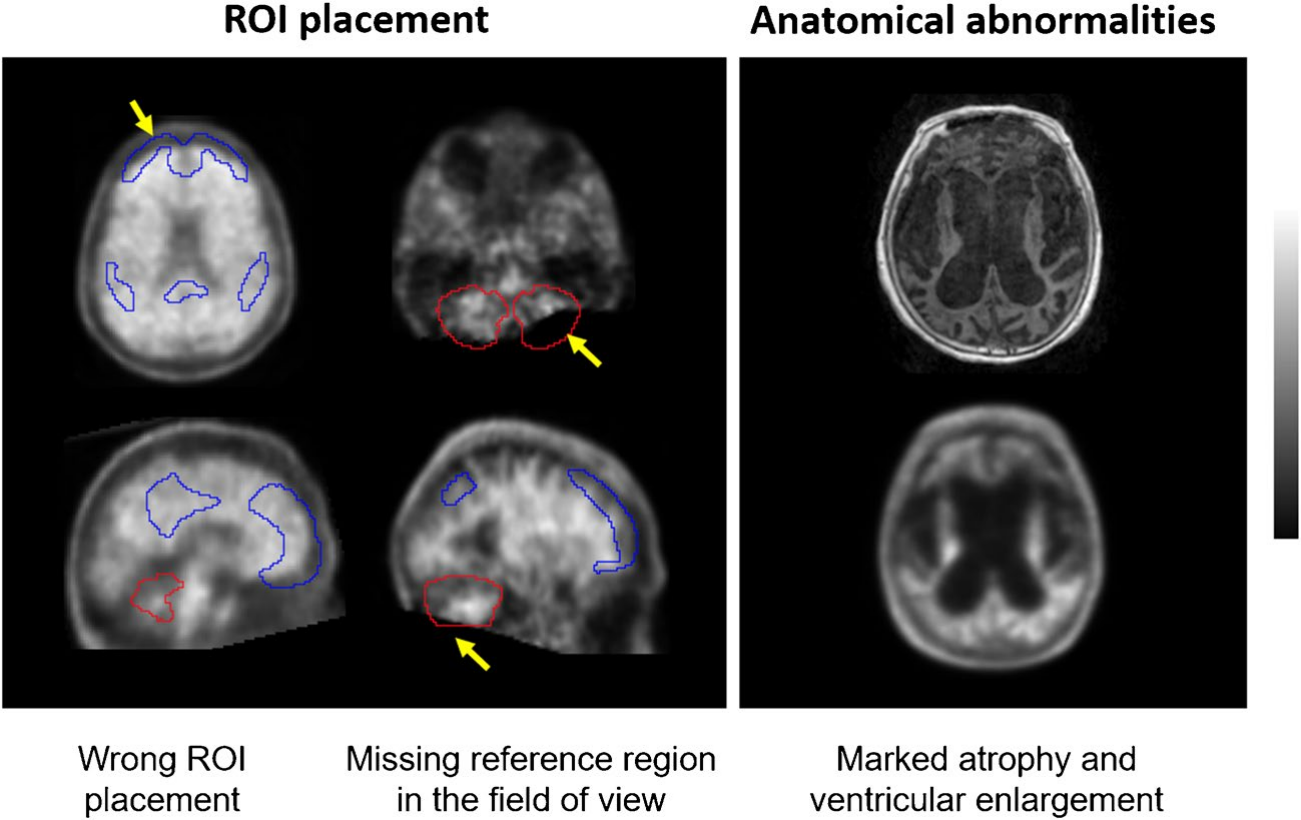
Examples of quality control issues that may impact the correct calculation of Aβ load from FBB PET scans. Left: yellow arrows indicate wrong cortical (blue) and reference region (red) ROI placement. Right: MRI (top) and FBB PET (bottom) scans illustrating marked atrophy and ventricular enlargement

All FBB PET scans were quantified with nine software packages using several metrics to estimate Aβ levels (Table 2). For some software packages, different analytical methods were tested using different reference regions or without using the T1-weighted MRI scan. All the scans were quantified in batch mode to minimize operator intervention. The operators were different for each software package and blinded to the diagnosis of subjects, demographics, visual PET assessment, histopathology results, and all other clinical data. All the results were quality controlled using the guidelines described in the supplementary material. In scans where quality control was not successful, the operator was allowed to use the available tools of the software to overcome quality control issues and to improve quantification. Figure 1 illustrates quality control issues that may impact the correct calculation of Aβ quantity from FBB PET scans. Subjects for whom quality control (QC) issues could not be resolved were excluded from the analysis of the individual analytical method (details and results of the QC procedure are described in the supplement). The quantification methods were assessed based on their continuous output, and binary classification of the continuous results was only used when comparing quantification to visual assessment.

**Table 2:** Summary of analytical methods included in the retrospective analysis

Analytical method id	Software	MRI required	Reference region	Metric	CE-marked	510(k)	References
#1	MIMneuro (v.7.1.2)	No	WC	CL	Yes	Yes	[30]
#2	Hermes BRASS (v.5.1.1)	No	WC	SUVR	Yes	Yes	
#3	Neurocloud (v.1.4)	No	WC	SUVR	Yes	No	
#4	Standard centiloid	Yes	WC	CL	No	No	[29, 31]
#5	Neurology Toolkit	No	WC	CL	No	No	[32]
#6	Neurology Toolkit	No	NA	Amyloid index	No	No	[32]
#7	SPM8	Yes	WC	SUVR	No	No	
#8	SPM8	Yes	WC*	SUVR	No	No	
#9	SPM8	Yes	CGM	SUVR	No	No	
#10	SPM8	No	WC	CL	No	No	
#11	PMOD Neuro (v.3.7)	Yes	CGM	SUVR	No	No	
#12	CapAIBL	No	WC	CL	No	No	[33–35]
#13	NMF CapAIBL	No	WC	CL	No	No	[36]
#14	Amyloid^IQ^	Yes	NA	Amyloid load	No	Submitted^1^	[37, 38]
#15	Amyloid^IQ^	No	NA	Amyloid load	No	Submitted^1^	[37, 38]

*CGM* cerebellar gray matter, *CL* centiloid, *NA* not applicable, *NMF* non-negative matrix factorisation, *SPM* statistical parametric mapping, *SUVR* standardized uptake value ratio, *WC* whole cerebellum (volume weighted average of CGM and WC), WC* whole cerebellum (average of CGM and cerebellar WM); ^1^personal communication

The non-commercial SPM-based methods using the T1-weighted MRI (analytical methods #7, #8, and #9) used spatial processing (PET-MRI registration and normalization to the standard template) according to the procedure specified by Klunk et al. [31]. ROIs were defined as the intersection between the standard automated anatomic labeling (AAL) atlas and the normalized gray matter segmentation map thresholded at a probability level of 0.2 [14]. The PET-only SPM-based method (analytical method #10) used the SPM8 tools to normalize each FBB PET scan on the standard space with an adaptive template generated from the average of several AD patients (*n* = 53) and amyloid-beta negative healthy controls (*n* = 55). The standard CL ROIs were applied to the normalized scan.

### Statistical methods

#### Sensitivity and specificity

The diagnostic performance of FBB PET scan analysis of visual and quantitative assessment was evaluated by means of sensitivity, specificity, accuracy, and their 95% confidence interval using histopathological confirmation as SoT (cohort #1). The cut-off for the established Aβ pathology for each analytical method was derived using receiver operating characteristic (ROC) curve analysis to ascertain the optimal threshold for the sensitivity and specificity calculation of the sample with histopathology confirmation of Aβ. The quantitative value that provided the highest Youden index (*J* = sensitivity + specificity − 1) was selected as the cut-off.

#### Concordance between visual and quantitative assessments

The percent agreement between visual read and binary quantitative assessments and the exact two-sided 95% Clopper-Pearson confidence interval in subjects from cohort #2 in subsets 1 and 2 were calculated for each reader assessment and the majority read (i.e., agreement of the majority of five readers). The concordance was also assessed in the subset of scans for which all the five independent blinded readers provided the same classification (“consensus” subset, subset 3).

#### Inter-software reliability

The reliability of agreement across software packages (i.e., inter-rater reliability) for the binary assessment (normal/abnormal Aβ load) in cohort #2 in subset 2 was performed by means of the Fleiss’ kappa (*κ*) and correlation analysis calculated across all pairs of analytical methods.

#### Intra-software reliability

A randomly selected subset of scans (*n* = 84) of cohort #2 (subset 4) was analyzed twice. Reliability was assessed by means of scatter plots and determination coefficients (*R*^2^).

#### Comparison of quantitative analytical methods using a public dataset

A linear regression was fitted to the Aβ metrics for each analytical method and the published centiloid (CL_GAAIN_) results [29] from the publicly available GAAIN dataset (cohort #4) as follows (Aβ metrics = *α* CL_GAAIN_ + *β*, where *α* and *β* are the constants of the model.

#### Distribution of Aβ load in elderly cognitively normal subjects

Aβ metrics were assessed in elderly cognitively normal subjects from cohort #2 (subset 3) that were assessed as Aβ-negative by consensus by five independent blinded readers. Histograms of the different Aβ metrics were generated and the 95th percentile of the Aβ metrics was calculated for each analytical method.

#### Aβ deposition over time

A linear regression model was fitted to each subject’s data in cohort #3, Aβ metric = *α* · *t* + *β*, where Aβ metric is the metric used to assess Aβ load, *α* and *β* are the coefficients of the model, and *t* is the scan time in years. The annual Aβ load increase was obtained from *α*. Subsequently, the average annual Aβ metric increase (*α*) was tested statistically by means of a Wilcoxon rank sum test whether Aβ-positive subjects, as defined by pipeline specific cut-offs derived from the pathology sample, are in the process of accumulating Aβ (i.e., (*H*_0_: *α* = 0; *H*_1_: *α* > 0). Descriptive statistics are reported as arithmetic mean and sample standard deviation (mean ± SD).

#### Prognostic accuracy of quantitative assessment

Clinical follow-up was evaluated in MCI subjects from cohort #3 to assess conversion to AD. The proportion of subjects that progressed and not progressed to AD after 4-year clinical follow-up and 95% exact Clopper-Pearson confidence intervals was calculated in each group (i.e., Aβ-negative or Aβ-positive at baseline). Positive likelihood ratio (LR +) and negative likelihood ratio (LR −) were also calculated. Descriptive statistics are reported as arithmetic mean and sample standard deviation (mean ± SD).

## Results

More than 11,000 PET scan quantifications from 589 subjects were analyzed in this retrospective analysis. All scans underwent quality control to ensure correct positioning of regions of interest (see supplementary material); 97.8% (range 94.95–100%) of the cases of the full sample met the stringent quality control criteria and were included in the presented analyses.

### Sensitivity and specificity (cohort #1)

Optimal quantitative cut-offs for each analytical method were developed using ROC curve analyses and histopathological confirmation as SoT (cohort #1). Centiloid values above 35 CL (average cut-off across analytical methods reporting centiloids of 36.2 ± 5.5 CL) indicate established Aβ pathology corresponding to moderate and frequent neuritic plaques by neuropathology. In order to make a fair comparison, a cut-off based on pathology was utilized in this study, aligning with the established method of VA. It is important to note that the threshold for identifying early amyloid pathology is continually evolving and is often determined using a group of young healthy controls [14].

The mean sensitivity, specificity, and accuracy were 96.1 ± 1.6%, 96.9 ± 1.0%, and 96.4 ± 1.1%, respectively, across all quantitative methods (cohort #1). For those analytical methods reporting centiloids, the results were 96.1 ± 1.6%, 97.4 ± 1.2%, and 96.7 ± 1.2%, respectively. The positive predictive value (PPV) and the negative predictive value (NPV) in this study population across all quantitative methods were 98.1 ± 0.5% and 93.7 ± 2.3%, respectively. Analyses of additional SoTs and examining a subgroup of CE-marked methods produced comparable results (see supplemental Table 1). The mean sensitivity, specificity, and accuracy of VA were 97.0 ± 1.7, 92.6 ± 1.3, and 95.2 ± 0.5, respectively, when performed by expert readers and 94.5 ± 3.3, 75.0 ± 18.2, and 88.4 ± 5.4 respectively, when performed by newly trained readers. The PPV and NPV values for expert readers in this study population were 95.3 ± 0.7% and 95.3 ± 2.4%, respectively, while newly trained readers had values of 87.4 ± 8.2% and 93.5 ± 4.7% for PPV and NPV, respectively. In this challenging end-of-life population, the overall accuracy of VA performed by expert readers was similar to quantitative assessment, but significantly lower for visual assessment performed by newly trained readers (Mann–Whitney test; *p* = 0.001), as it was also reported earlier from this dataset [9].

### Concordance between visual and quantitative assessment (cohort #2)

The mean percentage of agreement between binary quantitative assessment and visual majority assessment on the cohort #2 dataset was 92.4 ± 1.5% (range 88.9–94.9%) (subset 1: excluding subjects with autopsy that were used to generate the cut-offs) or 92.5 ± 1.5% (range 89.2–94.8%) (subset 2: whole cohort). For the consensus dataset (subset 3: reads had consensus VA), the mean percentage agreement was 97.4 ± 1.3% (range 93.8–99.1%). Analysis of a subgroup of CE-marked methods produced comparable results (see supplemental material).

**Fig. 2:**
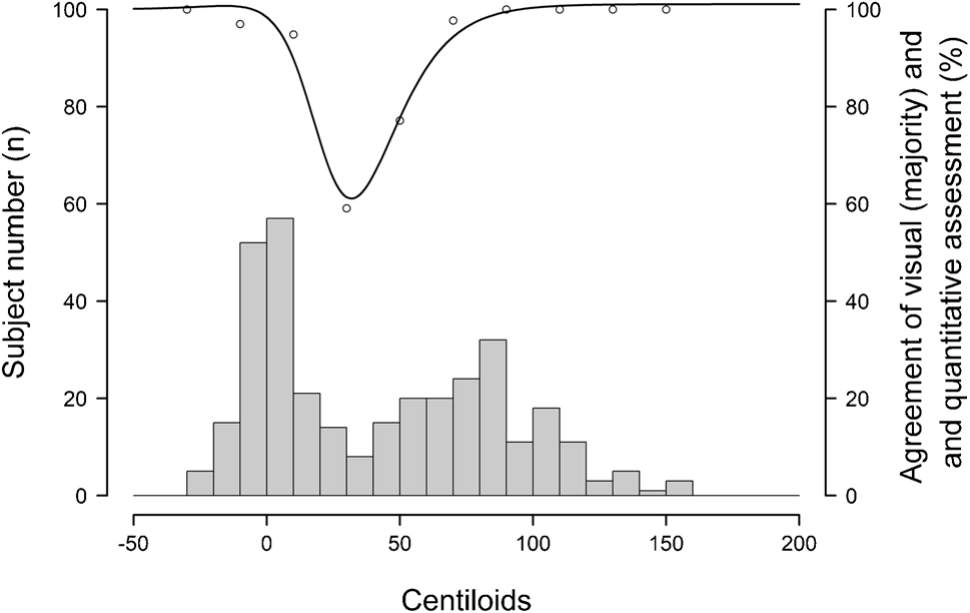
Agreement rate (circles) between visual and quantitative assessments (standard centiloid method). Histogram shows the subject numbers as a function of centiloid levels obtained from the standard centiloid method. The black line is the interpolation of the agreement rate. Concordance of the standard centiloid method with VA is 92.5% and is highest for cases with either absence or substantial presence of amyloid. Discordance between visual reads and quantification is highest at intermediate amyloid accumulation stages or close to pathology-derived cut-offs

Binarization of quantitative assessments was based on individual abnormality cut-offs derived from the pathology sample. Concordance between quantitative assessment and VA was highest for cases with either absence or substantial presence of amyloid. The agreement rate between methods dropped for cases with intermediate amyloid accumulation or close to pathology-derived cut-offs (see Fig. 2).

### Inter- and intra-software reliability (cohort #2)

Substantial agreement was observed across software packages. Fleiss’ *κ* for the inter-software reliability was 0.90 for the full cohort (cohort #2, subset 2), and 0.94 for the consensus cohort dataset (cohort #2, subset 3). The Fleiss *κ* of the VA across 5 independent blinded readers using the same subsets was 0.79 for the full cohort (cohort #2, subset 2). The inter-software agreement was also high for all possible software pairs ranging from 0.78 to 0.99 (cohort #2, subset 2). All pairs of analytical methods were linearly associated with an average correlation coefficient of 0.95 ± 0.03 (mean ± SD) ranging between 0.875 and 0.997 (cohort #2, subset 2).

The *R*^2^ value for all analytical methods’ re-analysis (intra-software reliability) ranged between 0.98 and 1.00 (cohort #2, subset 4).

### Comparison of quantitative analytical methods using a public dataset (cohort #4)

All the software methods that reported centiloids passed the validation criteria described by Klunk et al. (i.e., slope will be between 0.98 and 1.02, the intercept between  − 2 and 2 CL, and the *R*^2^ > 0.98.) [31]. Quantification results of all analytical methods were also tightly correlated with the centiloid values published in the literature [29], with determination coefficients *R*^2^ ranging from 0.92 to 0.99 for pipelines that do not report CLs. The *R*^2^ of the standard centiloid pipeline was 1.00, as expected. Other pipelines reporting centiloids that do not follow exactly the procedure described in Klunk et al. [31] had *R*^2^ values ranging from 0.98 to 0.99.

### Distribution of quantitative metrics in elderly healthy control subjects (cohort #2)

**Fig. 3:**
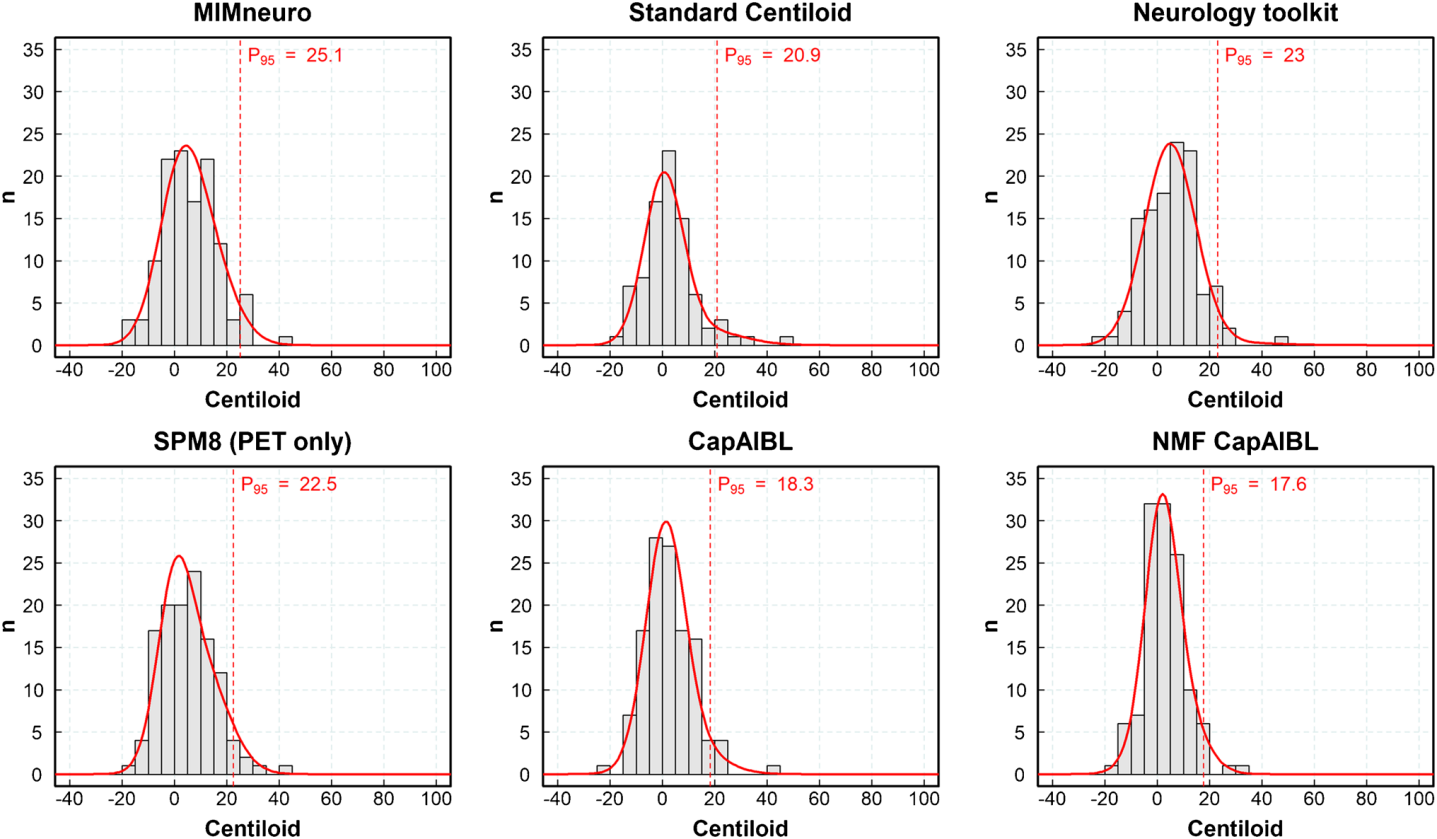
Distribution of CL values in elderly cognitively normal subjects with amyloid-beta negative visual assessment for different analytical methods and 95th percentile. The figure includes, for illustrative purposes, the Gaussian mixture model obtained from the sum of 2 Gaussian functions fitted to the data

The distribution of Aβ metrics was assessed in a subset of cognitively normal controls (*n* = 122 for PET-only analytical methods; *n* = 88 for analytical methods using T1-weighted MRI) from cohort #2, subset 3, who were classified by consensus as Aβ-negative by the five independent blinded readers. Table 3 lists the median and the 95th percentile of Aβ metrics calculated for each analysis method. The average 95th percentile for the six analytical methods that provided centiloid estimates was 21.2 ± 2.9 CL (mean ± SD, *n* = 6). Figure 3 shows the corresponding distribution histograms for centiloid methods.

**Table 3:** Median and 95th percentile of Aβ metrics calculated in elderly healthy controls (*n* = 122 for PET-only analytical methods; *n* = 88 for analytical methods using T1-weighted MRI) that were visually assessed as Aβ-negative by five independent blinded readers

Software	Metric	Median	95th percentile
#1 MIMneuro	Centiloid	5.01	25.09
#2 Hermes BRASS	SUVR	1.17	1.29
#3 Neurocloud	SUVR	1.08	1.28
#4 Standard centiloid	Centiloid	2.07	20.94
#5 Neurology Toolkit	Centiloid	5.56	22.97
#6 Neurology Toolkit	Amyloid index	− 0.44	− 0.30
#7 SPM8 (WC)	SUVR	1.09	1.21
#8 SPM8 (WC*)	SUVR	0.88	0.98
#9 SPM8 (CGM)	SUVR	1.18	1.33
#10 SPM8-PET only (WC)	Centiloid	4.57	22.52
#11 PMOD Neuro	SUVR	1.18	1.32
#12 CapAIBL	Centiloid	1.81	18.32
#13 NMF CapAIBL	Centiloid	2.95	17.59
#14 Amyloid^IQ^ (PET only)	Amyloid load	18.30	28.14
#15 Amyloid^IQ^ (MR)	Amyloid load	16.30	24.36

*CGM* cerebellar gray matter, *NMF* non-negative matrix factorisation, *SPM* statistical parametric mapping, *SUVR* standardized uptake value ratio, *WC* whole cerebellum (volume weighted average of CGM and WC), WC* whole cerebellum (average of CGM and cerebellar WM)

### Impact of amyloid status on (non)progression from MCI to AD dementia (cohort #3)

Patients in cohort #3 underwent repeated clinical evaluations over a period of up to 55 months after an initial baseline assessment [28]. On average across all quantification methods, 86.7 ± 4.1% of the Aβ-positive subjects by quantitative assessment at baseline progressed to AD dementia as revealed at the subsequent clinical follow-up. 1.7 ± 3.1% of Aβ-negative scans at baseline did progress to AD dementia. This relates to a positive likelihood ratio (LR +) of 8.2 ± 2.6 and a negative likelihood ratio (LR −) 0.02 ± 0.04.

The AD progression numbers based on VA at baseline were very similar. On average, 80.9 ± 5.8% of the Aβ-positive patients progressed to AD dementia within 55 months clinical follow-up. 1.9 ± 2.6% of subjects with a negative scan at baseline did progress to AD dementia. This relates to a positive likelihood ratio (LR +) of 5.3 ± 1.8 and a negative likelihood ratio 0.02 ± 0.03.

**Fig. 4:**
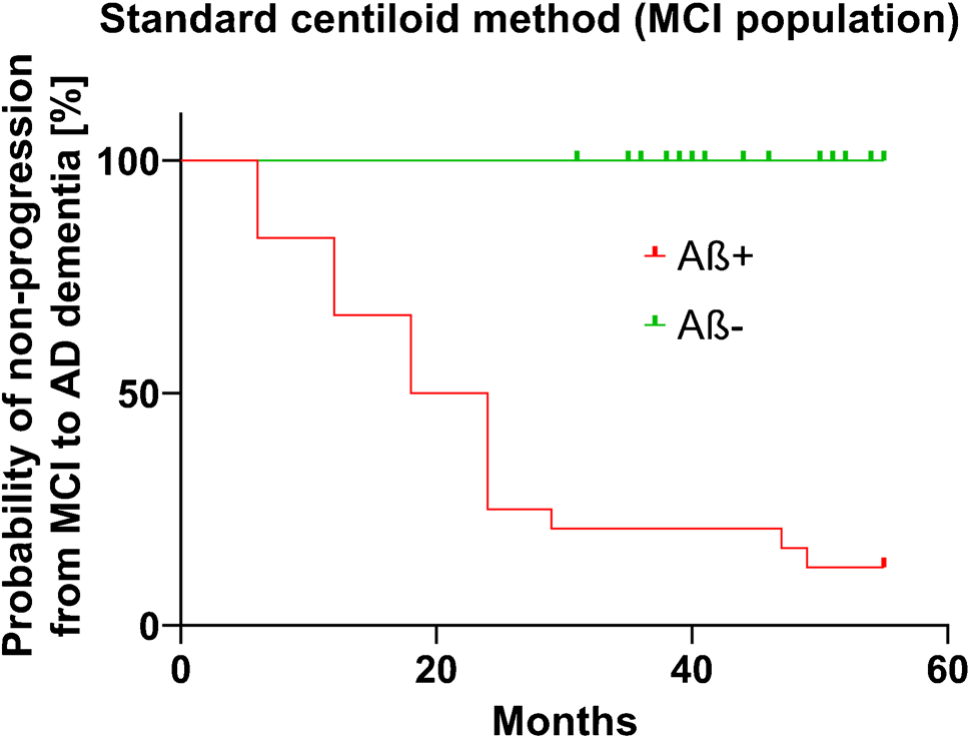
Kaplan-Meier survival analysis based on amyloid status assessed by the standard centiloid quantification method in an MCI population (cohort #3). Aß positivity is defined by the pathology-derived cut-off specific for the standard centiloid method

Positive Aβ scans in an MCI population were associated with significant increase in risk of clinical progression, as shown exemplarily for standard centiloid (Fig. 4).

### Assessment of Aβ deposition over time (cohort #3)

Amyloid-beta accumulation was assessed in MCI subjects with longitudinal FBB PET scans (cohort #3). The annual Aβ increase was estimated with a linear regression model fitted to each subject’s data. For all 15 analytical pipelines, subjects with established Aβ deposition at baseline had annual rates of Aβ accumulation statistically different from zero (*p* < 0.05). The average rate of CL increase per year in centiloid pipelines (*n* = 6) was 3.49 ± 1.19 CL for Aβ-positive subjects at baseline and 0.68 ± 0.77 CL for Aβ-negative subjects at baseline.

## Discussion

The results of this comprehensive analysis show that all investigated software quantification methods generate homogeneous and robust data and that quantification methods can complement visual assessment of FBB PET images. Adjunct use of quantification tools could be beneficial for newly trained or inexperienced operators, in instances when images are assessed with relatively low confidence based on visual assessment alone, to detect early amyloid deposition, or when amyloid levels of patients are close to “pathology” thresholds.

### Application of Aβ PET quantification

Excellent diagnostic efficacy of FBB PET quantification was demonstrated when compared to histopathology assessment as SoT. Mean sensitivity and specificity for all the software packages were 96.1% and 96.9%, respectively. The VA results suggest that less-experienced readers will likely benefit (in terms of improved specificity) of adjunct information obtained from quantitation, especially with difficult cases when the reader may have decreased confidence in the assessment. Similarly, the added value of adjunct use of quantification has been demonstrated for other amyloid tracers. Pontecorvo et al. suggested that access to quantitative information of the scans can improve the performance and confidence of some readers of florbetapir PET scans, particularly inexperienced readers [13]. Similarly, Kim et al. reported for florbetaben PET that when VA is supported by adjunct quantification, confidence in VA and inter-reader agreement is improved [39]. Finally, Bucci et al. reported that while quantitation of flutemetamol PET shows generally high agreement with VA, discordant cases with quantitative amyloid positivity are more likely to progress to AD, suggesting that quantification may be a tool to potentially detect earlier pathologic deposition [40].

The dichotomous quantitative assessment of FBB PET had a very high agreement of  > 92% to the majority VA. This result is consistent with previously published FBB data where percent agreement was reported between 88 and 97% [9, 10, 30, 41–47]. The average concordance of quantification and VA was higher (97.4%) in the consensus subset where images were only included when all five independent readers provided the same visual assessment. Very high agreement rates between quantification and VA have previously been shown for all amyloid PET tracers [5]. It is worth noting that typically quantitative methods tend to have higher concordance rates with expert readers than with non-expert readers [48].

While some details of individual quantification methods may differ, the software evaluation revealed homogenous performances in the current study. All software packages achieved excellent diagnostic efficacy when compared to histopathology and high concordance with visual assessments. Fleiss’ kappa between analytical methods was almost perfect and superior to Fleiss’ kappa of VA (0.9 and 0.79, respectively). This substantial inter-software agreement confirms good reproducibility of the results independent of the quantification method. The pairwise kappa agreement analysis led to similarly high agreement rates. Furthermore, all the quantitative metrics provided by the different analytical tools showed a tight linear agreement with an average correlation coefficient between pairs of analytical methods of 0.95 ± 0.03. This substantial correlation between different methods substantiates the generalisability of the obtained results, and this was confirmed by an additional analysis with a publicly available dataset (GAAIN) in which quantification results from the evaluated methods correlated closely with the standard centiloid method published previously [29]. Intra-software reproducibility was excellent for all methods and correlation coefficients for the individual method re-analysis ranged between 0.98 and 1.00. Overall, the relatively heterogenous analysis approaches of the different methods yielded very homogenous and robust results.

While all approved [^18^F]-labeled amyloid PET tracers have been validated based on their performance in late-stage disease patients against histopathology [8, 49, 50], the performance of quantitative methods in earlier stages of disease is important for the clinical routine situation. This was tested in cohort #3 (MCI), and the results seem particularly noteworthy, as they allow direct assessment of PET quantification in patients with early disease. Positive Aβ MCI patients in this cohort were associated with significant increase in risk of clinical progression, while negative Aβ patients, defined by the pathology-derived cut-off specific to each pipeline, did not progress to AD. The results of the different quantification methods and visual assessment were comparable demonstrating very high reliability and reproducibility and in concordance to the previously reported values for FBB [7, 28, 51, 52]. This confirms that quantification and visual assessment of FBB PET scans in early AD stages provide important information to predict progression to AD in MCI subjects over a 4-year observation period. This data is in line with results of the other amyloid tracers [53–56].

For all 15 analytical pipelines, subjects with established Aβ deposition at baseline had annual rates of Aβ accumulation statistically different from zero. Thus, quantification of amyloid load enables the evaluation of increased amyloid accumulation over time in a sensitive, objective manner; this can be particularly useful for low amyloid level subjects at an early disease stage and may help to identify groups of subjects most likely to benefit from early disease detection and possible therapeutic intervention. Indeed, selection by amyloid PET imaging has been a requirement to participate in therapeutic trials for many years [3, 4, 19–24].

### Use of quantitative information to supplement visual assessment

The correct interpretation of quantitative values is important for the appropriate use of quantitation to supplement VA. Interpretation of quantitative information of Aβ burden is conventionally performed by comparing the values against software-specific abnormality cut-offs or comparing against the typical distribution of negative and positive scans.

**Fig. 5:**
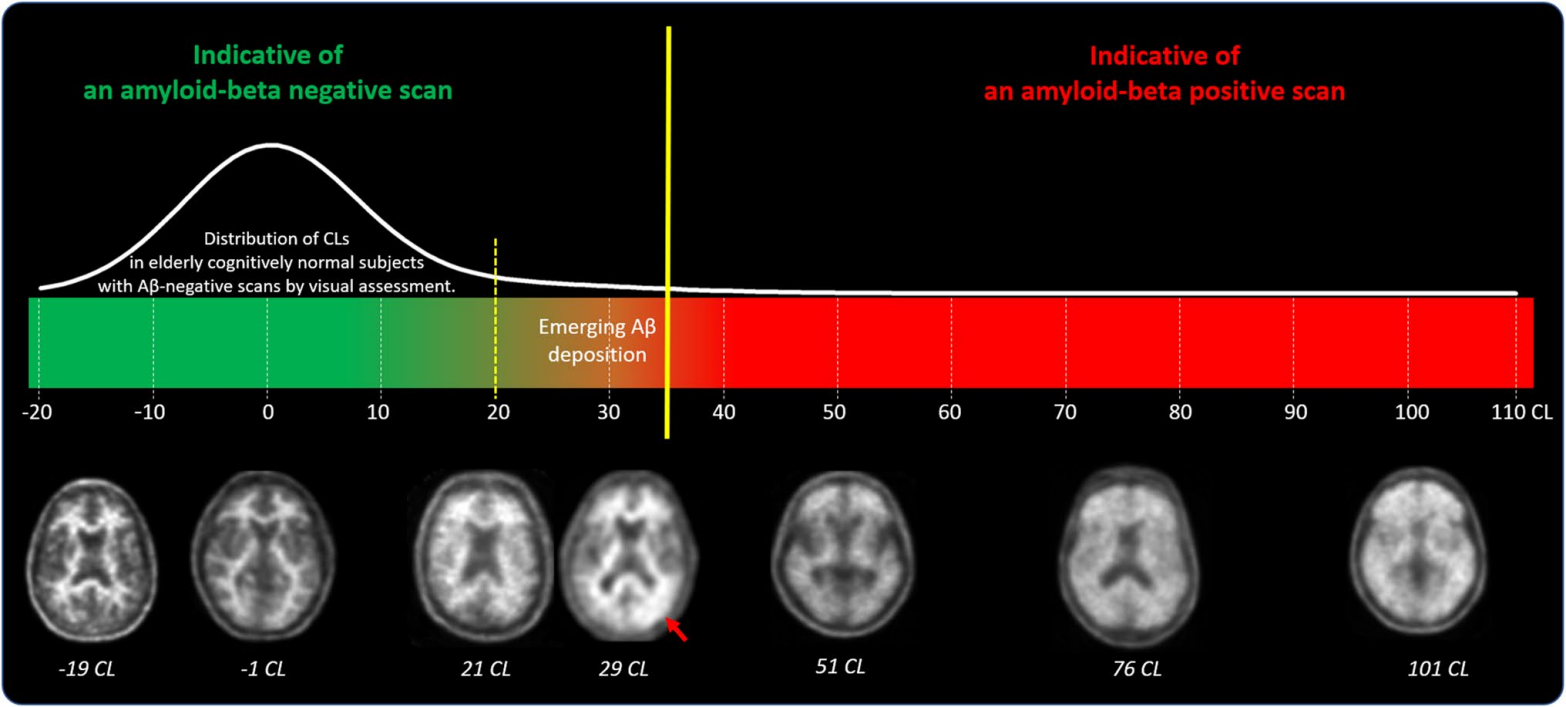
Illustrative display of the use of quantitative information to supplement visual assessment of FBB PET scans. Centiloid values above 35 CL indicate established Aβ pathology corresponding to a density of moderate and frequent neuritic plaques by neuropathology. Centiloid values below 20 represent elderly cognitively normal subjects with negative amyloid-beta scans by visual assessment. Centiloid values in the range between 20 and 35 CL are more likely to be ambiguous, can be either negative or positive by visual assessment, and correspond to subjects with emerging Aβ deposition. The readers should review such scans carefully to identify subtle amyloid accumulation that can be focal and/or unilateral (red arrow)

Those software packages that provided centiloid values reported similar cut-offs and interpretation of the results. Centiloid values above 35 CL indicate established Aβ pathology corresponding to moderate and frequent neuritic plaques by neuropathology. Subjects above this cut-off have a high probability of moderate or frequent plaques. Likewise, the agreement between VA and quantitative methods was extremely high for subjects with substantial amyloid levels. A lower cut-off of around 20 CL derived from elderly Aβ-negative, cognitively normal subjects provided a high specificity to rule out amyloid pathology. As for subjects with substantial amyloid deposition, agreement between VA and quantitative methods was very high for low amyloid levels. The level of agreement between VA and quantitative methods decreased for cases with intermediate amyloid accumulation or those near the pathology-derived cut-offs. This observation is consistent with the findings of Zeltzer et al., who reported a higher frequency of visual-quantitative discrepancies in scans near the amyloid positivity threshold [48]. Furthermore, subjects with CL values indicating intermediate amyloid deposition had a higher rate of not reaching full concordance between all 5 readers (see supplemental Fig. 1). These findings indicate that subjects with emerging or intermediate amyloid burden may need more careful inspection, benefiting from additional quantification derived information. Figure 5 is an illustrative example of the use of quantitative information to supplement visual assessment of FBB PET scans using centiloids.

**Fig. 6:**
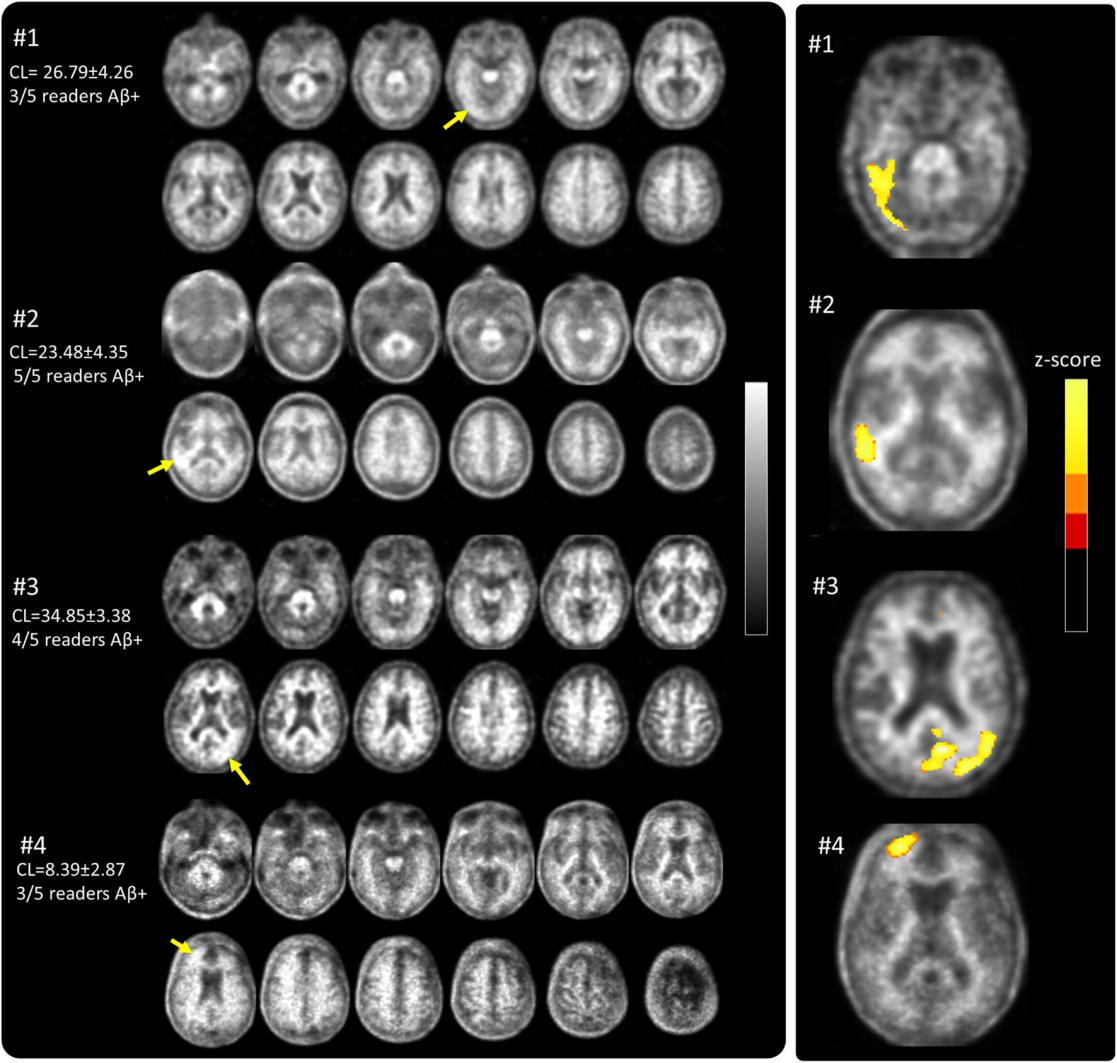
Illustrative examples of 4 cases (#1 to #4) that showed focal tracer uptake (arrow) and relatively low CL values (mean ± SD) obtained from the analytical pipelines analyzed. Also shown on the left is the number of independent blinded readers (out of 5 readers) that assessed the scans as positive (Aβ +). Focal tracer uptake with the corresponding *z*-scores is shown in the right panel. Only areas containing voxels with *z*-scores above 3 (in red, orange, and yellow color) are displayed. All 4 cases are clearly positive based on regional *z*-scores

As illustrated in Fig. 5, focal uptake is one factor that should be considered when interpreting global quantitative measures. Multiple studies and cohort types have demonstrated the robustness of centiloid quantification as a reliable measure [5, 52]. In the clinical implementation context, it is crucial to emphasize that the interpretation of quantitation should always be done in conjunction with a visual read. This ensures data quality, allows for understanding of possible causes of discordance, and helps prevent false-negative and false-positive classifications based solely on quantification or visual assessment. Figure 6 showcases examples of challenging cases characterized by emerging or focal amyloid deposition, which may result in discordance between visual readers and centiloid quantification. Most of the readers assessed these scans as positive, which were negative based on global binary quantitative assessments even though three of the cases present CL values pointing towards emerging Aβ pathology. In all these cases, regional information, e.g., as provided by *z*-score information, may provide additional incremental value and thus assist VA. As illustrated with these difficult and discordant patients in this work, focal uptake in regions less represented in the CL mask can result in lower quantitative values. In all these cases, regional information, e.g., as provided by *z*-score information, would provide an incremental value and thus assist correct classification of scans. This is in line with previous work [15, 26, 52, 57, 58].

The obtained centiloids of the current study agree with several recent reports across different tracers converging to the use of two cut-offs for amyloid PET abnormality: an early cut-off around CL = 11–20 where pathology may be emerging and a second around CL = 29–36 where amyloid burden levels correspond to moderate and frequent neuritic plaques. Bullich et al. developed a lower FBB PET cut-off of 13.5 CL when derived from young healthy controls indicating emerging Aβ pathology and derived a higher cut-off of 35.7 CL where amyloid burden levels correspond to established neuropathology findings [14]. The publication showed that these two cut-offs define a subset of subjects (13.5–35.7 CL) characterized by pre-AD dementia levels of amyloid burden that precede other biomarkers, such as tau deposition or clinical symptoms and accelerated amyloid accumulation. Other early cut-offs of 11, 14, or 17 CL have been reported for the FACEHBI, ALFA + , and AMYPAD PNHS (Prognostic and Natural History Study) studies using Gaussian mixture models [59]. Similarly, Salvadó et al. identified two cut-offs based on a direct comparison with established CSF Aβ42 thresholds: CL = 12 to rule out amyloid pathology and CL = 29 to denote established pathology [60]. Mormino et al. also showed the biological relevance of slight ^11^C-PIB elevations in elderly normal control subjects and provided an estimate for the cut-offs defining the “gray zone” using distribution volume ratios [17]. Using histopathological confirmation, Doré et al. and La Joie et al. reported gray zones from 12.2 to 24.4 and 19 to 28 CLs, respectively [61, 62]. Although this study achieved tightly correlated results between pipelines, especially between the different centiloid methods, it should be highlighted that even for the different centiloid approaches, a range of different cut-offs was observed, which is in line with previous reports that investigated the sensitivity of centiloid quantification to pipeline design [63, 64]. The variability in the cut-offs is not attributable to random test-retest errors, but to differences in pipeline-specific factors such as spatial processing, ROI definition, and SUVR to CL calibration bias. One centiloid pipeline used in the manuscript, the standard centiloid, adopts the approach developed by Klunk et al. (2015) and employs SPM8 for PET-MRI coregistration and normalization on the standard template, followed by the use of standard centiloid ROIs [31]. However, the other centiloid pipelines may employ different spatial processing algorithms and ROI definitions, leading to increased variability beyond the expected test–retest variability and resulting in different cut-offs for each pipeline. Additionally, the number of subjects excluded by quality control measures was low but slightly varied between pipelines, possibly leading to further increases in variability.

### Limitations

As a study limitation, an end-of-life population was used to estimate sensitivity and specificity with histopathological confirmation as SoT. This advanced population is characterized by anatomical abnormalities such as marked atrophy or ventricular enlargement, which hinders quantification and can pose a challenge for VA. Despite these challenges, the diagnostic efficacy of quantitative and VA was comparably good or even slightly higher for quantification in certain situations (i.e., for inexperienced readers).

Another limitation of the present study is that only indirect evidence on the adjunct value of quantification to visual assessment is provided. Such additional value can only be demonstrated in a prospective study. Furthermore, we acknowledge that an end-of-life cohort is less representative for early disease stages with lower amyloid burden. The derived sensitivity, specificity, and accuracy both for quantitative methods and readers were based on histopathology derived from such an end-of-life cohort, and these numbers should be considered in this context. However, similar or even better quantification performance is expected in an earlier study population, as fewer structural brain abnormalities are typically observed that could interfere with the assessments.

This study focused on simple binary reads for VA, which is the routine clinical method. However, recent research has shown the potential significance of amyloid PET regional quantification in staging AD [26, 27, 65], determining the risk of subsequent cognitive decline [27, 53], for optimal patient selection for anti-amyloid intervention trials [65, 66], and for reducing the sample size in anti-amyloid intervention trials [67, 68]. Pascoal et al. also showed that the topographical pattern of the PET signals in individuals with MCI who progress to dementia is “traditionally AD-like,” while that of non-converters includes more temporal and occipital regions instead [69]. The use of CL and composite SUVR is limited in determining the topographical distribution of Aβ load. Regional information may provide additional information to supplement visual assessment, but it has not been assessed in this analysis and is not widely available in all the quantitative tools. Such topographical information of Aβ load may enable earlier identification of subjects in the early-stage AD pathological continuum and may overcome simple dichotomous measures [15].

Finally, this study has also assessed global measurement of Aβ load such as SUVR, centiloids, amyloid load, or amyloid index. Some amyloid PET analysis software provides additional tools, such as *z*-scores obtained when comparing FBB PET signal from a scan with a normal database, which may assist with the clinical diagnosis. Such tools may offer additional information to supplement VA but were not evaluated in this study. Figure 6 presents several cases where regional *z*-score information may provide incremental value and thus assist VA.

## Conclusions

Visual binary reads of amyloid load provide the essential information for clinical routine diagnosis of AD but do not consider the wealth of information that brain PET scans provide. This study demonstrated that quantitative methods, using both CE marked software and other widely available processing tools, perform very homogenously and robustly, providing comparable results to visual assessments of FBB PET scans. Adjunct use of quantification could be beneficial in certain situations, e.g., for newly trained or inexperienced readers, in instances when images are visually assessed with relatively low confidence, for the early detection of amyloid load or when amyloid levels are close to “pathology” thresholds, for which assessment based on VA can be difficult. Physicians should retain the careful visual inspection of the images, but quantification methods may provide additional insights in cases of doubt or for research purposes.

### Supplementary Information

Below is the link to the electronic supplementary material.

Supplementary file1 (PDF 328 KB)

## Data Availability

Data can be made available for research purposes upon reasonable request.
