# Supplementary material for: Validation of quantitative assessment of florbetaben PET scans as an adjunct to the visual assessment across 15 software methods
Source: Eur J Nucl Med Mol Imaging. 2023 Jun 10;50(11):3276–89. doi: 10.1007/s00259-023-06279-0 (PMC10542295; doi:10.1007/s00259-023-06279-0)
Supplement: Supplementary file 1 — Supplementary file1 (PDF 328 KB) [file 259_2023_6279_MOESM1_ESM.pdf]

Validation of quantitative assessment of florbetaben PET scans as an adjunct to the visual assessment across 15 software methods

Author information

Aleksandar Jovalekic[1], Núria Roé-Vellvé[1], Norman Koglin[1], Mariana Lagos Quintana[1], Aaron Nelson[2], Markus Diemling[3], Johan Lilja[3], Juan Pablo Gómez-González[4], Vincent Doré[5], Pierrick Bourgeat[6], Alex Whittington[7], Roger Gunn[7], Andrew W. Stephens[1], Santiago Bullich[1]

[1]Life Molecular Imaging GmbH, Berlin, Germany, [2]MIM Software Inc., Cleveland, OH, USA, [3]Hermes Medical Solutions, Stockholm, Sweden, [4]Qubiotech Health Intelligence, A Coruna, Spain, [5]Department of Molecular Imaging & Therapy, Austin Health, Melbourne, Australia, [6]CSIRO, Brisbane, Australia, [7]Invicro, London, United Kingdom

Methods

Post-mortem histopathology

Post-mortem histopathological confirmation of Aβ- presence or absence in the brain was available for subjects from cohort #1. Besides the reported BSS/IHC assessment (SoT 1), two additional SoTs (SoT 2 and SoT 3) were applied in this retrospective study. SoTs 1 and 2, as described below, were based on the consensus histopathology assessment. Whereas, SoT 3, as described below, was based on the onsite histopathology assessment.

Consensus histopathology

The histopathology consensus panel classified neuritic beta-amyloid plaque density for Bielschowsky silver staining (BSS) according to CERAD scoring criteria (i.e., none, sparse, moderate, and frequent). A brain region was considered to have 'relevant amyloid-beta present', if the consensus panel judged it as having a final rating of "moderate" or higher. From the neuropathology consensus panel assessment of individual brain regions, a whole brain SoT was generated. A brain was classified as positive if amyloid-beta neuritic plaques were more than sparse in any of the six regions assessed. The subject was classified as negative if none of the six regions assessed were assessed as being more than sparse amyloid-beta neuritic plaques.

BSS is, however, not specific for beta-amyloid and in addition has technical limitations that may result in a lower detection sensitivity for neuritic plaques [1]. In fact, the combination of BSS with immunohistochemistry (IHC) for beta-amyloid staining is recommended in current neuropathology guidelines for assessment of Alzheimer's disease pathology [2, 3] to ensure the best histopathological detection sensitivity for neuritic plaques. For this reason, results from the regional BSS according to CERAD were combined in original histopathology study with the results from IHC staining (BSS/IHC) of the same brain regions, see [4, 5]. IHC assessment was scored with the same scoring system described for BSS. A region was considered positive if it was positive either by BSS or IHC. A subject was classified as positive if amyloid-beta neuritic plaques were more than sparse in any of the six regions assessed according to BSS/IHC. The subject was classified as negative if none of the six regions assessed were assessed as being more than sparse amyloid-beta neuritic plaques according to BSS/IHC. SoTs 1 and 2 were based on the consensus histopathology assessment.

Onsite histopathology

The onsite neuropathological diagnosis classified the brain specimens into 'relevant amyloid-beta present' no/yes. For this, the CERAD rating was applied according to Mirra et al. [6]. SoT 3 was based on the onsite histopathology assessment.

In this retrospective study, the primary efficacy analysis estimating the sensitivity and specificity was performed using three different whole brain standards of truth (SoT) as reported in the phase 3 histopathology study [4, 5]:

SoT 1: Consensus panel assessment of whole brain BSS according to CERAD in combination with IHC (BSS/IHC)

SoT 2: Consensus panel assessment of whole brain BSS according to CERAD (BSS)

SoT 3: Onsite neuropathology whole brain assessment based on CERAD

The determination of the specificity in cohort #1 was enriched by including ten young healthy volunteers (HVs). For these HVs no histopathological confirmation was possible, and the SoT was set to 'amyloid-beta not present' by definition for all brain regions.

The detailed findings using SoT 1 (BSS/IHC) is reported in the main paper. All three SoTs yielded similar results and are in full agreement and results on SoT 2 and SoT 3 are reported below.

PET quantification – quality control

Florbetaben PET scans (cohorts #1-4) were quantified with nine software packages using several metrics to estimate Aβ load (SUVR) [7], Centiloid [8, 9], amyloid load [10, 11] and amyloid index [12]. For some software packages, different analytical methods were tested using different reference regions. All the scans were quantified in batch mode to minimise operator (software user) intervention. The operators were different for each software package and blinded to all data pertaining to the subject. After image processing, all the results were quality controlled using the following guidelines:

1) Correct placement of the cortical regions of interest (ROIs) on the brain image was assessed. Cortical ROIs must be placed on the grey matter regions and should not have included cerebrospinal fluid (CSF) or significant areas of white matter.
2) Correct placement of the reference region ROIs to ensure that these are well fitted to the region was assessed.
3) Where applicable, the correct co-registration of MRI and florbetaben PET scans was assessed.
4) In subjects where ROIs were derived from the segmentation of the MRI, the correct segmentation of the cortex was visually assessed.
5) In the analytical pipelines that did not use ROIs (e.g., Amyloid^{IQ}, Neurology toolkit), the quality control included the assessment of the correct spatial normalisation of the PET scans with canonical images or templates.

In those scans, where quality control was not successful, the operator was allowed to use the available tools of the software to correct quantification. Subjects that did not pass quality control were excluded from the individual software package analysis.

<u>Results</u>

Sensitivity and specificity

The procedure to classify florbetaben PET scans by visual and quantitative assessment was evaluated by means of sensitivity, specificity and accuracy and their 95% confidence interval using histopathological confirmation as SoT (cohort #1). Optimal quantitative cut-offs for each analytical pipeline were developed using ROC curve analyses and histopathological confirmation as SoT. Those software packages that used similar ROIs or calibrated their metrics to centiloids reported similar cut-offs.

As reported in the main manuscript, using SoT 1 the mean sensitivity, specificity and accuracy were 96.1±1.6%, 96.9±1.0% and 96.4±1.1%, respectively, for all quantitative methods. Results obtained using additional SoTs or for CE-marked software packages only produced comparable data that is presented in the Supplemental table 1.

Supplemental table 1: Sensitivity, specificity and accuracy of quantitative assessment of florbetaben PET scans for all analytical pipelines and CE-marked pipelines using different histopathology SoTs.

	Quantitative assessment (mean±SD)	Quantitative assessment (CE-marked) (mean±SD)
SoT 1 (BSS/IHC) (Sensitivity)	96.1±1.6	95.8±1.8
SoT 1 (BSS/IHC) (Specificity)	96.9±1.0	98.1±1.4
SoT 1 (BSS/IHC) (Accuracy)	96.4±1.1	96.7±1.6
SoT 2 (BSS) (Sensitivity)	95.3±2.2	94.6±3.1
SoT 2 (BSS) (Specificity)	87.0±3.2	90.1±1.4
SoT 2 (BSS) (Accuracy)	92.0±1.0	92.6±1.1
SoT 3 (onsite) (Sensitivity)	93.4±1.5	93.8±1.5

SoT 3 (onsite) (Specificity)	93.3±1.5	94.0±2.4
SoT 3 (onsite) (Accuracy)	93.4±.9	93.9±.9

Concordance between visual and quantitative assessment

The mean percentage of agreement between binary quantitative assessment and visual majority assessment on the cohort #2 dataset was high for all subset analysis (see **Supplemental table 2**).

Supplemental table 2: Percent agreement between quantitative and majority visual assessment

	Quantitative assessment (mean±SD)	Quantitative assessment (CE-marked) (mean±SD)
Full sample (excluding subjects from histopathology study used to generate the cut-off)	92.4±1.5	91.2±1.7
Consensus sample (readers had consensus in VA, i.e., all 5 readers independently assessed the scans in the same way)	97.4±1.3	96.2±1.8

Reader agreement across the continuum of amyloid load

The scans in which the readers could not reach a consensus were not uniformly spread across the continuum of amyloid load, as determined by quantification. Such reader disagreement in their assessments was more prevalent in borderline cases and near the established cutoff for amyloid-beta pathology. The figure below provides a representation of how visual disagreement is distributed across the amyloid continuum, in this example for the standard centiloid method. Subjects with CL values ranging from 25 to 50 CL had a 62% rate of not reaching full concordance between all 5 readers. Outside this range, the discordant cases were between 0-20%. While atrophy can make visual assessments more challenging, this effect was mainly notable in borderline cases. However, the readers were able to accurately interpret cases with low or high amyloid levels even in the presence of atrophy. Identifying subtle uptake that is unilateral and localized in a single region in the early population is difficult and can decrease consensus between readers. The clinical population’s diagnosis did not significantly impact consensus between readers.

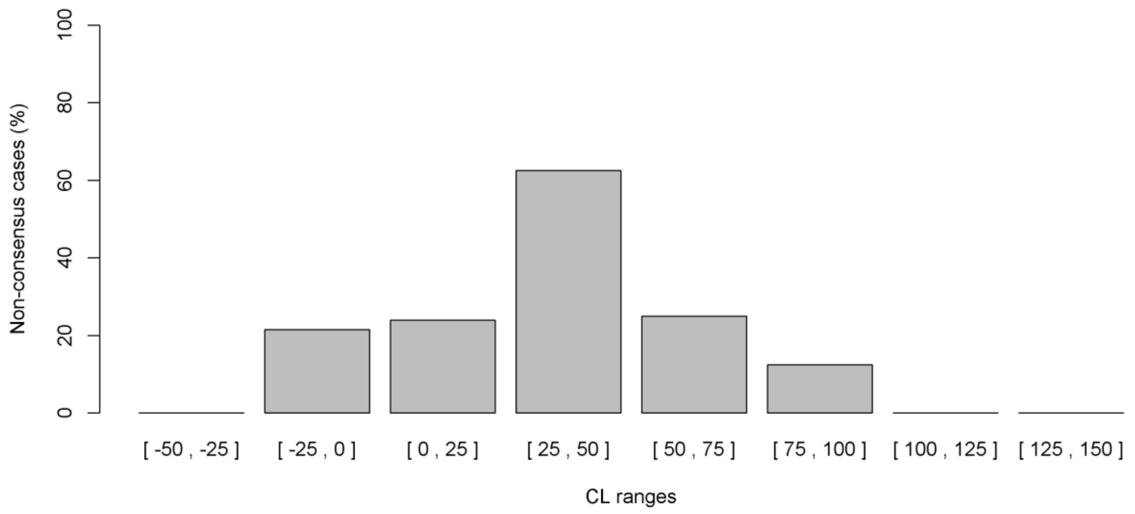

Supplemental figure 1: Percent of cases for which five readers did not reach full consensus. The underlying sample (n=336, quality controlled cohort #2, subset 2) is presented for the “standard centiloid” MR-based pipeline.

Inter-software reliability

Substantial agreement of the software packages was observed, with a kappa estimate of 0.89 (95% CI: 0.86, 0.92) in subset 1, which is the dataset excluding subjects from the histopathology study (i.e. those used to generate the cut-offs). A kappa value of 0.90 (95% CI: 0.88, 0.93) was obtained across all analytical pipelines when analyzing subset 2, which is the dataset including subjects from the histopathology study. A kappa value of 0.94 (95% CI: 0.92, 0.97) was obtained for subset 3, which is the consensus dataset (i.e., all blinded readers assessed the scans in the same way).

The Fleiss' kappa across of 5 independent blinded readers in the same dataset without and with subjects from the study were 0.80 (95% CI: 0.76 – 0.84) and 0.79 (95% CI: 0.75 – 0.83), respectively.

Supplemental table 3: Fleiss' kappa (and confidence interval) across analytical pipelines and VA.

	**Fleiss' kappa (quantification pipelines)**	**Fleiss' kappa (visual assessment)**
Full sample (excluding subject from histopathology study used to generate the cut-off)	0.89 (0.86, 0.92)	0.80 (0.76, 0.84)
Consensus sample (readers had consensus in VA, i.e., all 5 readers independently assessed the scans in the same way)	0.94 (0.92, 0.97)	1.00 (1.00, 1.00)

References supplementary material

1. Uchihara, T., *Silver diagnosis in neuropathology: principles, practice and revised interpretation.* Acta Neuropathol, 2007. **113**(5): p. 483-99.
2. Montine, T.J., et al., *National Institute on Aging-Alzheimer's Association guidelines for the neuropathologic assessment of Alzheimer's disease: a practical approach.* Acta Neuropathol, 2012. **123**(1): p. 1-11.
3. Hyman, B.T., et al., *National Institute on Aging-Alzheimer's Association guidelines for the neuropathologic assessment of Alzheimer's disease.* Alzheimers Dement, 2012. **8**(1): p. 1-13.
4. Sabri, O., et al., *Florbetaben PET imaging to detect amyloid beta plaques in Alzheimer's disease: phase 3 study.* Alzheimers Dement, 2015. **11**(8): p. 964-74.
5. Seibyl, J., et al., *Impact of Training Method on the Robustness of the Visual Assessment of 18F-Florbetaben PET Scans: Results from a Phase-3 Study.* J Nucl Med, 2016. **57**(6): p. 900-6.
6. Mirra, S.S., et al., *The Consortium to Establish a Registry for Alzheimer's Disease (CERAD). Part II. Standardization of the neuropathologic assessment of Alzheimer's disease.* Neurology, 1991. **41**(4): p. 479-86.
7. Becker, G.A., et al., *PET quantification of 18F-florbetaben binding to beta-amyloid deposits in human brains.* J Nucl Med, 2013. **54**(5): p. 723-31.
8. Klunk, W.E., et al., *The Centiloid Project: standardizing quantitative amyloid plaque estimation by PET.* Alzheimers Dement, 2015. **11**(1): p. 1-15 e1-4.
9. Rowe, C.C., et al., *(18)F-Florbetaben PET beta-amyloid binding expressed in Centiloids.* Eur J Nucl Med Mol Imaging, 2017. **44**(12): p. 2053-2059.
10. Whittington, A., et al. *AmyloidIQ – an automated algorithm for classifying amyloid-β PET scans*. in *Human Amyloid Imaging 2019*. 2019.
11. Whittington, A., R.N. Gunn, and I. Alzheimer's Disease Neuroimaging, *Amyloid Load: A More Sensitive Biomarker for Amyloid Imaging.* J Nucl Med, 2019. **60**(4): p. 536-540.
12. Leuzy, A., et al., *Derivation and utility of an Abeta-PET pathology accumulation index to estimate Abeta load.* Neurology, 2020. **95**(21): p. e2834-e2844.
